# Large Language Model Versus Manual Review for Clinical Data Curation in Breast Cancer: Retrospective Comparative Study

**DOI:** 10.2196/73605

**Published:** 2025-11-06

**Authors:** Young-Joon Kang, Hocheol Lee, Jae Pak Yi, Hyobin Kim, Chang Ik Yoon, Jong Min Baek, Yong-seok Kim, Ye Won Jeon, Jiyoung Rhu, Su Hyun Lim, Hoon Choi, Se Jeong Oh

**Affiliations:** 1 Department of Surgery, College of Medicine, The Catholic University of Korea, Incheon St Mary's Hospital, 56, Dongsu-ro, Bupyeong-gu, Incheon, 21431, Republic of Korea, 01026383847; 2 Department of AI Health Information Management, Yonsei University (Mirae), Wonju, Republic of Korea; 3 Department of Surgery, College of Medicine, The Catholic University of Korea, Seoul St Mary's Hospital, Seoul, Republic of Korea; 4 Department of Surgery, College of Medicine, The Catholic University of Korea, Yeouido St Mary's Hospital, Seoul, Republic of Korea; 5 Department of Surgery, College of Medicine, The Catholic University of Korea, Uijeongbu St Mary's Hospital, Uijeongbu, Republic of Korea; 6 Department of Surgery, College of Medicine, The Catholic University of Korea, St Vincent's Hospital, Suwon, Republic of Korea; 7 Department of Surgery, College of Medicine, The Catholic University of Korea, Bucheon St Mary's Hospital, Bucheon, Republic of Korea

**Keywords:** natural language processing, breast neoplasms, data mining, clinical oncology, large language model, artificial intelligence

## Abstract

**Background:**

Manual review of electronic health records for clinical research is labor-intensive and prone to reviewer-dependent variations. Large language models (LLMs) offer potential for automated clinical data extraction; however, their feasibility in surgical oncology remains underexplored.

**Objective:**

This study aimed to evaluate the feasibility and accuracy of LLM-based processing compared with manual physician review for extracting clinical data from breast cancer records.

**Methods:**

We conducted a retrospective comparative study analyzing breast cancer records from 5 academic hospitals (January 2019-December 2019). Two data extraction pathways were compared: (1) manual physician review with direct electronic health record access (group 1: 1366/3100, 44.06%) and (2) LLM-based processing using Claude 3.5 Sonnet (Anthropic) on deidentified data automatically extracted through a clinical data warehouse platform (group 2: 1734/3100, 55.94%). The automated extraction system provided prestructured, deidentified data sheets organized by clinical domains, which were then processed by the LLM. The LLM prompt was developed through a 3-phase iterative process over 2 days. Primary outcomes included missing value rates, extraction accuracy, and concordance between groups. Secondary outcomes included comparison with the Korean Breast Cancer Society national registry data, processing time, and resource use. Validation involved 50 stratified random samples per group (900 data points each), assessed by 4 breast surgical oncologists. Statistical analysis included chi-square tests, 2-tailed *t* tests, Cohen κ, and intraclass correlation coefficients. The accuracy threshold was set at 90%.

**Results:**

The LLM achieved 90.8% (817) accuracy in validation analysis. Missing data patterns differed between groups: group 2 showed better lymph node documentation (missing: 152/1734, 8.76% vs 294/1366, 21.52%) but higher missing rates for cancer staging (211/1734, 12.17% vs 43/1366, 3.15%). Both groups demonstrated similar breast-conserving surgery rates (1107/1734, 63.84% vs 868/1366, 63.54%). Processing efficiency differed substantially: LLM processing required 12 days with 2 physicians versus 7 months with 5 physicians for manual review, representing a 91% reduction in physician hours (96 h vs 1025 h). The LLM group captured significantly more survival events (41 vs 11; *P*=.002). Stage distribution in the LLM group aligned better with national registry data (Cramér V=0.03 vs 0.07). Application programming interface costs totaled US $260 for 1734 cases (US $0.15 per case).

**Conclusions:**

LLM-based curation of automatically extracted, deidentified clinical data demonstrated comparable effectiveness to manual physician review while reducing processing time by 95% and physician hours by 91%. This 2-step approach—automated data extraction followed by LLM curation—addresses both privacy concerns and efficiency needs. Despite limitations in integrating multiple clinical events, this methodology offers a scalable solution for clinical data extraction in oncology research. The 90.8% accuracy rate and superior capture of survival events suggest that combining automated data extraction systems with LLM processing can accelerate retrospective clinical research while maintaining data quality and patient privacy.

## Introduction

### Background

Recent advances in artificial intelligence, particularly in large language models (LLMs), have demonstrated remarkable capabilities of automated data extraction and organization from complex clinical documents [1-3]. These artificial intelligence–driven approaches can be used to process large volumes of clinical data with a consistent methodology, potentially reducing human bias and improving the collection efficiency of research data. Although LLMs show promise in health care applications, few studies on their practical efficacy compared with that of traditional, manual processing by physicians have been published, particularly in complex areas such as the extraction of surgical oncology data [4].

### Clinical Challenges in Cancer Data Curation

In the field of breast cancer surgery, retrospective data analysis presents unique challenges owing to the complexity of unstructured clinical data. Electronic health records (EHRs) contain diverse information across clinical charts, operation records, and pathology reports, often in a free-text format. The complexity is compounded by breast cancer–specific characteristics, including the bilateral nature of the organs, concurrent malignant and benign lesions, and multiple radiological features. This complicates automated data curation and has traditionally necessitated manual review by physicians for accurate data interpretation and collection.

However, this manual approach has several limitations. As the volume of clinical data increases, consistency in physician reviews becomes increasingly difficult to maintain, potentially leading to discrepancies in data interpretation [5,6]. The time-intensive nature of manual review and the risk of errors in the processing of large volumes of clinical data present considerable challenges in retrospective research [7-10]. In addition, direct EHR access for manual data extraction raises privacy concerns when sensitive patient information is handled [11-13]. Although LLM-based automation of information extraction from anonymized EHR data may address these challenges, its effectiveness compared with that of traditional physician reviews remains to be evaluated.

### Study Objectives

Although LLMs have been validated for the extraction of specific medical data, their potential for the curation of comprehensive data of patients with cancer remains largely unexplored [14]. In this study, we compared traditional physician reviews with LLM-based processing of anonymized clinical data in the field of breast cancer, focusing on the development of a practical approach for surgical oncologists. We hypothesized that LLM-based analysis would yield comparable results to manual review in handling large volumes of clinical data while reducing processing time and resource use.

## Methods

### Study Design and Data Collection

This retrospective comparative study included patients with breast cancer who underwent surgery at 5 academic hospitals from January 1, 2019, to December 31, 2019. This study was designed to compare 2 practical data curation pathways available in real-world clinical research settings. The manual review pathway represents the traditional method of direct EHR access. The LLM processing pathway uses preextracted, deidentified data. This study adheres to the Transparent Reporting of a Multivariable Prediction Model for Individual Prognosis or Diagnosis–Large Language Model (TRIPOD-LLM) reporting guidelines (Checklist 1).

We compared 2 data extraction methods: manual physician review (group 1) and LLM-based processing (group 2). In group 1, 1 dedicated breast-surgical oncologist from each hospital reviewed data spanning 2 years (2019‐2020) over 7 months (May 2021–November 2021) using a standardized data collection form (Multimedia Appendix 1). The data encompassed 89 clinical variables across 3 domains: patient demographics (basic information, medical history, and family history), treatment information (surgical details, neoadjuvant or adjuvant therapy, complications, and follow-up treatment), and pathological information (tumor characteristics, tumor stage, biomarker status, and margin status). Follow-up observations regarding recurrence and mortality were updated until January 2024.

Patients in group 2 were initially identified using the clinical data warehouse (CDW) of Catholic Medical Center, an integrated data platform of 8 affiliated academic hospitals in Korea [15,16]. The CDW supports this research by providing anonymous clinical data to investigators following institutional review board approval [15]. The LLM structured 31 clinical factors from the raw data, including patient demographics (basic information, survival data, and diagnostic data), treatment information (surgery types and neoadjuvant or adjuvant therapy), pathological information (tumor characteristics, tumor stage, biomarker status, and nodal status), and imaging features. Data extraction and curation were performed from October 20, 2024, to November 1, 2024.

The CDW query identified 17,317 patients diagnosed with invasive breast cancer or ductal carcinoma in situ between July 2018 and July 2021. From this cohort, we selected patients diagnosed during the study period (January 2019-December 2019) who underwent breast cancer surgery. CDW extraction included unstructured EHR reports containing clinical information, operation records, and pathology reports (MultimediaAppendices 2^4^). Follow-up data through October 31, 2023, were used.

### Data Curation in LLM-Processing Group

Unstructured data extracted from the CDW were processed using Claude 3.5 Sonnet (Anthropic) to extract and structure the required factors into predefined categories.

#### LLM Implementation and Application Programming Interface (API) Access

We accessed Claude 3.5 Sonnet through the Anthropic web interface (claude.ai) using a professional subscription account. The implementation specifications are as follows:

1. Access method: web-based interface with manual copy-paste of clinical documents.
2. Processing approach: sequential processing of individual patient records.
3. Input size limitations: documents exceeding 100,000 characters were split into logical sections (diagnosis, surgery, and pathology) and processed sequentially.
4. Output format: structured CSV format directly generated by the LLM.
5. Session management: new conversation initiated for each batch of 50 patients to prevent context contamination.
6. Quality control: real-time review of outputs with immediate reprocessing for any parsing errors.

No API programming or authentication keys were required, as we used the standard web interface. This approach, while manual, ensured direct oversight of the extraction process and immediate error detection.

#### Prompt Development Process

##### Overview

The LLM prompt was developed through a 3-phase iterative process over 2 days (from October 20, 2024, to October 21, 2024). Rather than manually crafting the extraction rules, we used an interactive dialogue approach with the LLM itself to develop the prompt. We provided the LLM with sample data and target output requirements, and then iteratively refined the extraction protocol through conversational feedback.

The iterative refinement process consists of 3 phases.

##### Phase 1

This was the initial framework development phase and we included 10 cases. We presented the LLM with representative raw data and developed extraction rules through dialogue. The LLM proposed initial patterns for data extraction, which were tested using sample cases.

##### Phase 2

This was the rule refinement phase, and we included 20 cases. On the basis of the phase 1 outputs, we organized errors through manual review and engaged LLM to analyze the errors and modify them. Key refinements included diagnosis deduplication using International Classification of Diseases, 10th Revision code comparison, surgical procedure hierarchy establishment, pathology section prioritization, and biomarker interpretation standardization (particularly for human epidermal growth factor receptor 2 [HER2] status requiring in situ hybridization confirmation for 2+ cases).

##### Phase 3

This was the edge case handling phase, and we included 30 cases. The refined prompt was tested in diverse clinical scenarios. Additional instructions were added for handling bilateral cases (processing each breast separately), multiple surgical procedures (capturing all relevant operations), ambiguous staging information (requiring explicit notation rather than inference), and complex biomarker patterns (particularly HER2 equivocal cases).

The final prompt is available in the Multimedia Appendix 5. Due to its length, we provide a condensed version highlighting the key extraction rules, while the complete prompt with all edge cases and examples can be obtained from the corresponding author.

### Prompt Structure and Components

The final prompt focused on accurate extraction from 4 distinct data categories: diagnostic information, clinical measurements, surgical procedures, and pathological findings.

#### Global Processing Rules Module

This module established standardized data formats and processing conventions. Key specifications included patient ID formatting (R[9-digit number]), laterality coding (right or left or bilateral), date standardization (YYYY-MM-DD), missing data coding (999,999), and CSV output structure. Each case was processed by laterality to handle bilateral breast cancers as separate entities, maintaining distinct Case_IDs formatted as “Patient_ID_Laterality” (eg, R000000001_RT).

#### Clinical Data Processing Module

This module handled diagnosis information, surgical procedures, and mortality data with specific extraction hierarchies. For diagnosis processing, the system prioritized primary diagnosis information, extracted English text only while removing Korean text, and implemented deduplication logic by comparing International Classification of Diseases, 10th Revision codes (first three characters) to identify identical diagnoses while preserving distinct diagnoses. For surgical information, we established clear precedence rules where therapeutic operations took priority over diagnostic procedures, with specific terminology mapping for breast procedures (eg, “wide excision,” “modified radical mastectomy,” and “lumpectomy”) and axillary procedures (sentinel lymph node biopsy [SLNB] and axillary lymph node dissection [ALND]).

#### Pathology Data Extraction Module

Given the variability in pathology report formats, we defined a section priority hierarchy: (1) microscopic description, (2) diagnosis, (3) immunohistochemistry, and (4) gross description. Specific extraction patterns were defined within each section. For tumor size determination, the search sequence was: “tumor size (size of largest invasive carcinoma)," “greatest dimension of largest invasive focus,” “size of largest invasive focus,” and “estimated size (extent) of DCIS.” For lymph node assessment, we standardized various reporting formats (eg, “X/Y [positive/total]," “metastatic carcinoma [n/total]," “lymph node metastasis; present or absent [n/total]") and classified metastasis by size (macrometastasis >2 mm, micrometastasis 0.2‐2 mm, and isolated tumor cell ≤0.2 mm).

#### Quality Control Module

this module implemented validation rules and error prevention strategies. The prompt excluded error-prone extractions, such as gross specimen sizes, surgical margin measurements, and lymph node sizes. It required explicit documentation of missing data rather than inference, implemented range validation for biomarkers (estrogen receptor or progesterone receptor or Allred score 0‐8, Ki-67 0%‐100%), and cross-reference verification between different report sections.

### Task Sequence and Processing Flow

Prompt execution of tasks in a specific sequence to ensure data integrity:

1. Initial parsing: identify patient ID and laterality from diagnostic records
2. Temporal alignment: establish diagnosis date as a reference point for all subsequent data
3. Hierarchical extraction: process data in order of clinical importance: diagnosis → surgery → pathology → imaging
4. Integration check: validate consistency across different data sources
5. Output generation: structure extracted data into a predefined .CSV format with quality flags

The prompt instructed the LLM to generate outputs directly in .CSV format with predefined column structures, automated date formatting (YYYY-MM-DD), and standardized missing data codes (999,999), eliminating the need for extensive after processing. The structured output was validated through the methodology described in the Data Quality Assessment and Validation section.

### Objectives and Statistical Analysis

This study aimed to assess the feasibility of replacing manual physician reviews with LLM-based processing of breast cancer–related clinical data. We compared the demographic characteristics, clinical parameters, treatment patterns, disease characteristics, and survival outcomes between the 2 groups.

Categorical variables were compared using chi-square or Fisher exact tests, with agreement assessed using Cohen κ coefficient (κ<0.20=poor, 0.21‐0.40=fair, 0.41‐0.60=moderate, 0.61‐0.80=good, >0.80=very good). Continuous variables were analyzed using the Student *t* test and the intraclass correlation coefficient. Effect sizes were calculated using Cohen *d* (continuous) and Cramér V (categorical).

Overall survival was analyzed using the Kaplan-Meier method and compared using the log-rank test. Both approaches were validated using the Korean Breast Cancer Society (KBCS) 2019 national registry data by comparing age, tumor stage, surgical procedures, molecular subtypes, and survival trends [17].

### Data Quality Assessment and Validation

For validation, 50 cases from each group were selected using proportionate stratified random sampling. Stratification was based on the cancer stage (0-IV) and type of surgical intervention (breast-conserving surgery vs mastectomy) to ensure representative sampling across key clinical categories. Random selection was performed using Python (version 3.8; Python Software Foundation) with the *NumPy* (v.1.21.0) and *pandas* (v.1.3.0) libraries and a fixed random seed of 2,02,41,201 for reproducibility. Four breast-surgical oncologists (SJO, JPY, HK, and SL) independently evaluated 18 predefined clinical factors in each case (900 data points per group). Accuracy rates were calculated as the percentage of correctly extracted factors relative to the total number of factors. A dual-reference validation approach was implemented: group 1 was validated against the EHR, whereas group 2 was compared to the CDW raw data. The evaluation included both present and missing values. The accuracy threshold was set at 90% based on previous validation studies of clinical data extraction systems [18].

### Ethical Considerations

This study was approved by the institutional review board of the Catholic Medical Center (approval: OC24WIDI0138). As this was a retrospective analysis of existing clinical data, the requirement for informed consent was waived by the institutional review board. All patient data were deidentified prior to analysis, with personal identifiers replaced by anonymized codes. The CDW platform ensures privacy protection through automated deidentification. No compensation was provided to participants, as this study involved retrospective data analysis only.

## Results

### Outcomes

**Figure 1.**
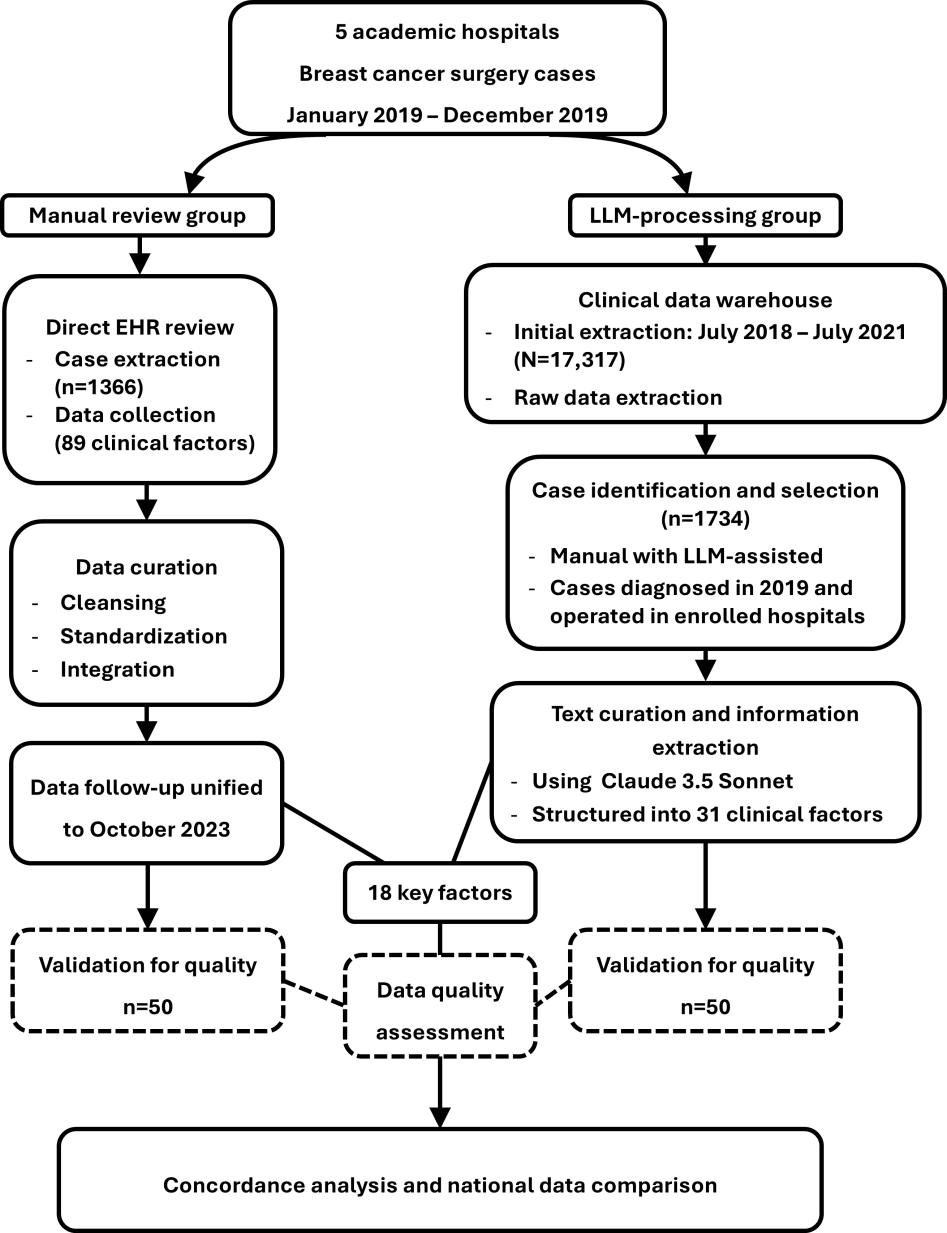
Study design chart showing the comparison between the manual review and large language model (LLM)-processing groups. EHR: electronic health record.

**Figure 2.**
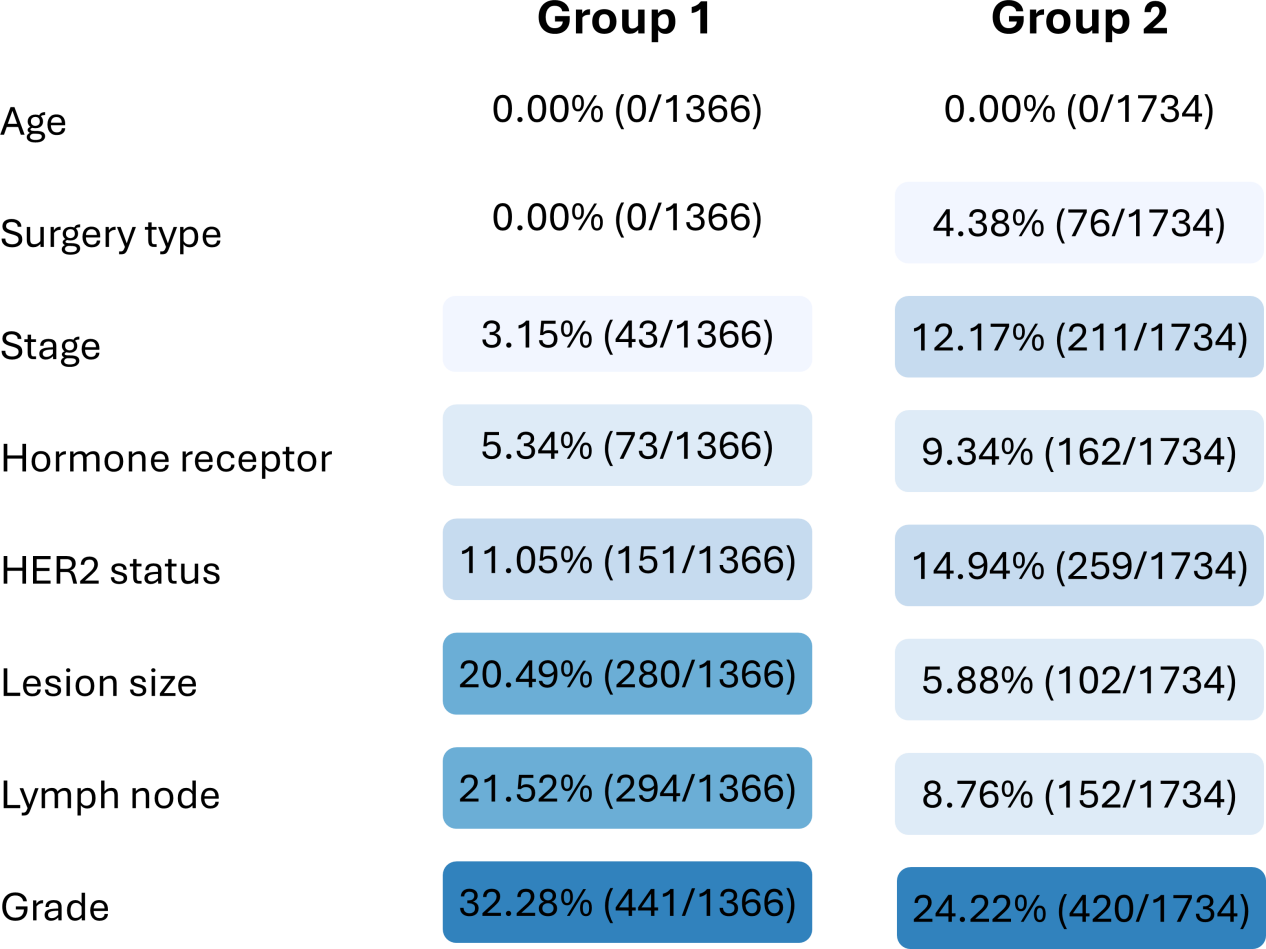
Comparison of missing data rates (%) between manual review (group 1) and large language model (LLM) processing (group 2). Color intensity represents the magnitude of missing data, with darker shades indicating higher missing rates. HER2: human epidermal growth factor receptor 2.

For comparative analysis, 18 key clinical factors were selected from both groups (Figure 1). The manual review (group 1) and LLM processing (group 2) groups comprised 1366 and 1734 cases, respectively. Although both groups completely captured age data, they exhibited different patterns of missing data for the other parameters. Group 2 had higher missing rates in terms of cancer stage (12.2% vs 3.1%) and HER2 status (15.1% vs 11.0%), whereas group 1 had more missing data for lesion size (20.5% vs 5.9%) and lymph node assessment (21.5% vs 8.8%). Both groups maintained high documentation rates (>90%) for hormone receptor status (Figure 2).

The validation analysis encompassed 1800 data points (900 per group) across clinical factors. Group 1 demonstrated perfect accuracy with no discrepancies. Group 2 exhibited 83 discordant factors out of 900 data points with an accuracy rate of (817/900, 90.8%). Among 1734 patients in group 2, 260 (15%) underwent multiple surgical procedures. The LLM successfully integrated data from sequential operations in 53% (138/260) of these cases, while missing data integration in 47% (122/260).

### Processing Time and Resource Use

LLM-based processing demonstrated efficiency gains compared to manual review (Table 1). Group 1 required 7 months (May 2021-November 2021) with 5 dedicated breast surgical oncologists. In contrast, group 2 processing was completed in 12 days (from October 20, 2024, to November 1, 2024) by 2 physicians, with data extraction taking 10 days and validation requiring 2 additional days.

**Table 1.** Comparison of processing efficiency.

Parameter	Manual review (group 1)	LLM^a^ processing (group 2)	Difference
Total cases	1336	1734	+368
Processing period	7 months	12 days	−95%
Number of physicians	5	2	−60%
Total physician hours	Approximately 1025	Approximately 96	−91%
Direct EHR^b^ access required	Yes	No	N/A^d^
API^c^ cost	N/A^d^	Approximately $260	N/A

a LLM: large language model.

b EHR: electronic health record.

c API: application programming interface.

d N/A: not applicable.

Manual review required approximately 1025 physician hours total across 7 months. LLM processing required approximately 96 physician hours in total over 12 days, a 91% reduction in time investment.

Resource use differed substantially between the methods. The manual review required 5 breast surgical oncologists with a direct EHR access infrastructure and dedicated data entry personnel. LLM processing required 2 physicians using a standard workstation without EHR access. The API cost for LLM processing was approximately $0.15 per case, totaling $260 for 1734 cases. Although direct personnel costs were not calculated due to institutional variations in physician compensation, the 91% reduction in physician hours represents substantial resource savings.

### Demographics and Clinical Characteristics

The baseline characteristics of both groups are summarized in Table 2. The mean age differed slightly between groups 1 and 2 (55, SD 11.5 vs 53.5, SD 11.4 y; *P*<.001; Cohen *d*=0.13). For breast surgery, total mastectomy was performed in 19.2% of cases in group 1 and 26.5% in group 2, while nipple (skin)-sparing mastectomy rates were 15.7% and 9.6%, respectively (*χ*^2_3_^=164.3; *P*<.001; Cramér V=0.29). When these procedures were combined, groups 1 and 2 exhibited similar proportions of breast-conserving surgery (63.5% vs 63.9%) and mastectomy (34.8% vs 36%), with a small effect size (Cramér V=0.10).

**Table 2.** Baseline characteristics of study groups. Statistical significance set at *P*<.05. Analyses were performed using chi-square test for categorical variables and *t* test for continuous variables^a^.

Characteristics	Manual review (n=1366)	LLM^b^ processing (n=1734)	*P* value
Demographics, mean (SD)			
Age (y)	55.0 (11.5)	53.5 (11.4)	<.001
Surgical procedures, n			
Breast operation			<.001
Breast-conserving surgery	63.53 (868/1366)	63.90 (949/1485)	
Total mastectomy	19.18 (262/1366)	26.46 (393/1485)	
N(S)SM^c^	15.67 (214/1366)	9.56 (142/1485)	
Other procedures	1.61 (22/1366)	0.07 (1/1485)	
Combined mastectomy^d^	34.85 (476/1366)	36.03 (535/1485)	
Axillary surgery			<.001
No surgery	11.20 (153/1366)	19.37 (321/1657)	
SLNB^e^	68.30 (933/1366)	59.75 (990/1657)	
ALND^f^	20.50 (280/1366)	20.88 (346/1657)	
Pathological results, mean (SD)			
Tumor size (mm)	20.5 (16.4)	21.5 (16.9)	.156
Lymph node status, mean (SD)			
Harvested nodes	7.79 (7.0)	7.11 (7.2)	.016
Metastatic nodes	0.95 (3.0)	0.98 (3.2)	.802
Stage distribution, n (%)			<.001
0	17.91 (237/1323)	15.56 (237/1523)	
IA	35.68 (472/1323)	43.27 (659/1523)	
IB	0.60 (8/1323)	1.31 (20/1523)	
IIA	22.22 (294/1323)	20.75 (316/1523)	
IIB	11.11 (147/1323)	10.83 (165/1523)	
IIIA	7.48 (99/1323)	5.65 (86/1523)	
IIIB	0.83 (11/1323)	0.07 (1/1523)	
IIIC	3.70 (49/1323)	2.56 (39/1523)	
IV	0.45 (6/1323)	0.00 (0/1523)	
Biomarker status			
ER^g^ positive, % (n/N)	78.28 (1012/1293)	76.21 (1198/1572)	.172
PR^h^ positive, % (n/N)	68.68 (886/1290)	67.50 (1059/1569)	.525
HER2^i^ positive, % (n/N)	20.49 (249/1215)	20.20 (298/1475)	.003
Ki-67, mean (SD)	25.4 (23.0)	26.6 (22.6)	.204
Histologic grade, % (n/N)			.764
Grade 1	22.81 (211/925)	21.54 (283/1314)	
Grade 2	44.65 (413/925)	46.35 (609/1314)	
Grade 3	32.54 (301/925)	32.12 (422/1314)	
Nuclear grade, % (n/N)			<.001
Grade 1	16.75 (171/1021)	12.90 (199/1543)	
Grade 2	42.51 (434/1021)	51.52 (795/1543)	
Grade 3	40.78 (416/1021)	35.58 (549/1543)	
Survival outcomes, % (n/N)			
Death	0.81 (11/1366)	2.42 (42/1734)	.001

a Percentages calculated based on cases with available data for each variable

b LLM: large language model.

c N(S)SM: nipple or skin-sparing mastectomy.

d Combined mastectomy includes total mastectomy and N(S)SM.

e SLNB: sentinel lymph node biopsy.

f ALND: axillary lymph node dissection.

g ER: estrogen receptor.

h PR: progesterone receptor.

i HER2: human epidermal growth factor receptor 2.

In terms of axillary surgery, group 1 had a higher rate of SLNB than group 2 (68.4% vs 59.7%), whereas the rates of ALND were similar (*χ*^2^_2_=47.2; *P*<.001; Cramér V=0.13). The mean (SD) value of the harvested lymph nodes was similar between the groups (7.79, SD 7.03 vs 7.11, SD 7.20; *P*=.016).

The stage distribution differed between groups (*χ*^2^_8_=68.9*; P*<.001), but only slightly (Cramér V=0.16), with group 1 identifying more cancers as advanced. Hormone receptor status was similar between the groups (estrogen receptor: 78.3% vs 76.2%, *P*=.172; progesterone receptor: 68.7% vs 67.5%, *P*=.525). HER2 status differed negligibly, and Ki67 expression was similar between the groups (HER2: *P*=.003, Cramér V=0.003; Ki67: *P*=.391, Cramér V was approximately 0.00). Histological grade distributions were similar between the groups (*P*=.764).

### Interrater Agreement Analysis

ICC analysis of continuous variables demonstrated a consistently low agreement: age (ICC 0.013, 95% CI –0.035 to 0.060), tumor size (ICC 0.029, 95% CI –0.021 to 0.078), number of metastatic lymph nodes (ICC 0.031, 95% CI –0.019 to 0.081), number of harvested lymph nodes (ICC 0.025, 95% CI –0.025 to 0.075), and Ki67 expression (ICC 0.027, 95% CI –0.023 to 0.077). All ICC values were negligible, and all CIs included zero.

### Survival Outcomes

Survival analysis revealed significant differences between the groups (hazard ratio 2.917, 95% CI 1.496 to 5.688; *P*=.002). Group 2 captured more events (11 vs 41). The proportional hazards assumption was met (χ²_₁_=2.37; *P*=.120), and the log-rank test confirmed a difference in survival distributions (χ²_₁_=10.9; *P*=.001).

### Comparison With National Registry Data

Comparison with the KBCS 2019 registry data (N=9447) revealed small differences in breast surgery patterns for both groups (Cramér V=0.03‐0.04; *P*≤.018; Table 3). For axillary surgery, both groups had lower SLNB rates (group 1: 68.30% and group 2: 59.75% vs KBCS: 73.18%) and similar ALND rates (20.50% vs 20.88% vs 18.60%). Group 2 had a higher rate of no axillary surgery than group 1 (19.37% vs 11.20%).

Stage distribution analysis revealed significant but small differences from the national data (group 1: Cramér V=0.076, *P*<.001; group 2: Cramér V=0.038, *P*=.003). Regarding biomarker subtypes, both groups had slightly higher proportions of hormone receptor–positive with HER2-negative (group 1: 67.02% and group 2: 66.57% vs KBCS: 63.14%) and triple-negative cases (12.70% and 13.59% vs 11.98%) with minimal effect sizes (Cramér V=0.03‐0.04).

**Table 3.** Comparison of clinical characteristics with Korean Breast Cancer Society (KBCS) 2019 national registry data^a^.

Characteristic	Group 1 (n=1366)	Group 2 (n=1734)	KBCS 2019 (N=9447)	Effect size (Cramér's V)	P value^b^
Breast surgery type, n (%)				0.03-0.04	<.001
Breast-conserving surgery	868 (63.53)	949 (63.90)	6067 (64.26)		
Total mastectomy	476 (34.85)	535 (36.03)	3380 (35.78)		
Others	22 (1.61)	1 (0.07)	0 (0.00)		
Axillary surgery type, n (%)				Group 1: 0.13 Group 2: 0.15	<.001
SLNB^d^	933 (68.30)	990 (59.75)	6913 (73.18)		
ALND^e^	280 (20.50)	346 (20.88)	1757 (18.60)		
No surgery	153 (11.20)	321 (19.37)	777 (8.22)		
Cancer stage distribution, n (%)				Group 1: 0.07 Group 2: 0.03	<.001 0.003
Stage 0	237 (17.91)	237 (15.56)	1588 (16.81)		
Stage I	480 (36.28)	679 (44.58)	4015 (42.50)		
Stage II	441 (33.33)	481 (31.58)	2948 (31.20)		
Stage III	159 (12.02)	126 (8.27)	896 (9.48)		
Biomarker status,^f^ n (%)				0.03-0.04	0.003
ER^k^ positive	1012 (78.28)	1198 (76.21)	7163 (75.82)		
PR^h^ positive	886 (68.68)	1059 (67.50)	6254 (66.20)		
HER2^i^ positive	249 (20.49)	298 (20.20)	1748 (18.50)		

a Percentages calculated based on cases with available data for each variable

b Statistical significance set at *P*<.05. Chi-square tests were used for categorical comparisons.

c KBCS: Korean Breast Cancer Society

d SLNB: sentinel lymph node biopsy.

e ALND: axillary lymph node dissection.

f Molecular subtypes were determined based on combined estrogen receptor (ER), progesterone receptor (PR), and HER2 status. HR+ defined as ER+ or PR+.

g ER: estrogen receptor.

h PR: progesterone receptor.

i HER2: human epidermal growth factor receptor 2.

## Discussion

### Principal Findings

This comparative study demonstrated that LLM-based processing achieved 90.8% accuracy in extracting clinical data from breast cancer records, with significant reductions in processing time (12 d vs 7 mo) and resource requirements (2 vs 5 physicians). The LLM approach captured substantially more survival events (41 vs 11; *P*=.002) and showed better documentation of lymph node assessment (91.2% vs 78.5%), although it had higher missing rates for integrated assessments such as cancer staging (12.2% vs 3.1%). Both methods yielded similar patterns in key clinical parameters, including breast-conserving surgery rates (63.5% vs 63.9%) and biomarker distributions.

A particular finding was the substantial difference in captured survival events between LLM processing (41 events) and manual review (11 events). This discrepancy may reflect fundamental differences in how humans and LLMs approach large-scale data extraction. Manual reviewers processing many charts may inadvertently adopt a mechanical approach, focusing on the most obvious data fields, while potentially overlooking mortality information scattered across multiple sections of the medical record. In contrast, LLM maintained consistent thoroughness throughout the extraction process, systematically examining all available data sources for each case without the cognitive fatigue that affects human reviewers during repetitive tasks.

This finding challenges the assumption that manual review is the gold standard for all types of clinical data extraction. While human expertise remains essential for complex clinical interpretation, our results suggest that LLM processing may provide a more complete capture of certain objective outcomes, particularly those requiring synthesis across multiple data fields. However, this interpretation requires further research to confirm whether the additional events captured by the LLM represent true positives or extraction errors.

### Comparison With Prior Work

Although LLMs have shown favorable results in extracting specific medical data from radiology and pathology reports [19-22], our study represents the first comprehensive evaluation of surgical oncology data curation. Previous studies have focused on the extraction of single data points or specific types of reports. For instance, Park et al [20] demonstrated the effectiveness of LLM in extracting pulmonary disease information from radiology reports, whereas Cheng [22] reviewed its applications in pathology. Our study extends these findings by demonstrating that LLMs can handle the complex integration required for comprehensive oncological data including surgical procedures, pathological findings, and survival outcomes.

The observed statistical differences between groups, while significant, were mostly clinically negligible (Cramér V<0.30 for all comparisons), suggesting that LLM processing maintains clinical validity. The observed accuracy of 90.8% is comparable to recent studies, where LLMs achieved similar performance in extracting structured information from clinical notes, including the social determinants of health [23]. Although comprehensive systematic reviews of LLM applications in healthcare are still emerging [3], individual studies have consistently demonstrated their potential for automated clinical data extraction. However, unlike previous studies that focused on extracting discrete clinical variables or single-domain information [19-23], surgical oncology data require integration of multiple interconnected factors, presenting unique challenges in automated extraction.

### Strengths and Limitations

Our study had several strengths. The large sample size provided robust statistical power. The use of real-world clinical data from multiple institutions enhanced the generalizability of the results. The head-to-head comparison with a manual physician review rather than against a reference standard alone provides practical insights for clinical research applications. Additionally, the demonstrated efficiency gains (12 d vs 7 mo) highlight the potential for scaling oncological research.

However, several limitations of this study must be acknowledged.

First, LLMs showed limitations in integrating multiple clinical events. Although the model performed well in extracting explicit data points, it struggled to synthesize information across sequential surgical procedures. This was evidenced by the higher rate of missing surgical data (12.2% vs 3.1%) in patients who underwent multiple operations. Manual reviewers could identify and integrate multiple surgical steps, such as lymph node assessment after initial diagnostic excision, while the LLM typically captured data from a single representative operation.

Second, the validation sample size of 50 cases per group represented only 2.9% and 2.6% of the respective cohorts. While this sample was stratified to ensure representation across cancer stages and surgical types and achieved adequate power for detecting clinically meaningful differences, a larger validation set would strengthen confidence in the accuracy estimates.

Third, differences in data sources between the groups may have affected direct comparability. The manual review group accessed complete EHRs directly, whereas the LLM group processed CDW-extracted data. This methodological design was unavoidable; manual reviewers needed direct EHR access for a comprehensive review, while the LLM required deidentified extracted data for processing. However, this means we cannot isolate whether performance differences stemmed from the extraction approach itself or from the inherent differences in available data. Future studies comparing both methods using identical raw data would overcome this limitation.

Fourth, generalizability to other LLM models requires evaluation. We used Claude 3.5 Sonnet, but the performance may vary across models, versions, and prompting strategies. The rapid evolution of LLM capabilities suggests that our findings represent a snapshot of current technology rather than definitive limits.

### Future Directions

Future research should address several key issues. The first is the development of improved prompting strategies to handle complex clinical scenarios that require integrated assessment, particularly for sequential surgical procedures and temporal relationships. Second, LLM processing is compared with manual review using identical raw data sources to isolate the actual performance differences between the methods. Third, feasibility studies of integrated data curation across multiple clinical events are needed to overcome the current limitations in synthesizing longitudinal patient data. Fourth, a systematic examination of the performance characteristics of different LLM models is required to identify optimal models for specific clinical data types.

### Conclusions

LLM-based processing demonstrated comparable effectiveness to manual review by physicians for breast cancer clinical data extraction, while significantly reducing processing time and resource utilization. Despite the limitations of integrated assessments requiring synthesis across multiple clinical events, this approach offers a solution for efficient clinical data extraction in oncology research. The ability to process large volumes of data consistently and rapidly while maintaining an accuracy above 90% suggests that LLM-based methods can accelerate retrospective clinical research.

## Supplementary material

10.2196/73605 Multimedia Appendix 1 Standardized data collection form used for manual physician review

10.2196/73605 Multimedia Appendix 2 Example of grouped data sheets extracted from the clinical data warehouse.

10.2196/73605 Multimedia Appendix 3 Pathology reports extracted from the clinical data warehouse

10.2196/73605 Multimedia Appendix 4 Radiology reports extracted from the clinical data warehouse.

10.2196/73605 Multimedia Appendix 5 The condensed prompt.

10.2196/73605 Checklist 1 TRIPOD-LLM checklist.

## Abbreviations

ALND: axillary lymph node dissection
API: application programming interface
CDW: clinical data warehouse
EHR: electronic health record
HER2: human epidermal growth factor receptor 2
ICC: intraclass correlation coefficient
KBCS: Korean Breast Cancer Society
LLM: large language model
SLNB: sentinel lymph node biopsy
TRIPOD-LLM: Transparent Reporting of a Multivariable Prediction Model for Individual Prognosis or Diagnosis–Large Language Model
