# Supplementary figures and images for: Large Language Model Versus Manual Review for Clinical Data Curation in Breast Cancer: Retrospective Comparative Study
Source: JMIR Med Inform. 2025 Nov 6;13:e73605. doi: 10.2196/73605 (PMC12599480; doi:10.2196/73605)

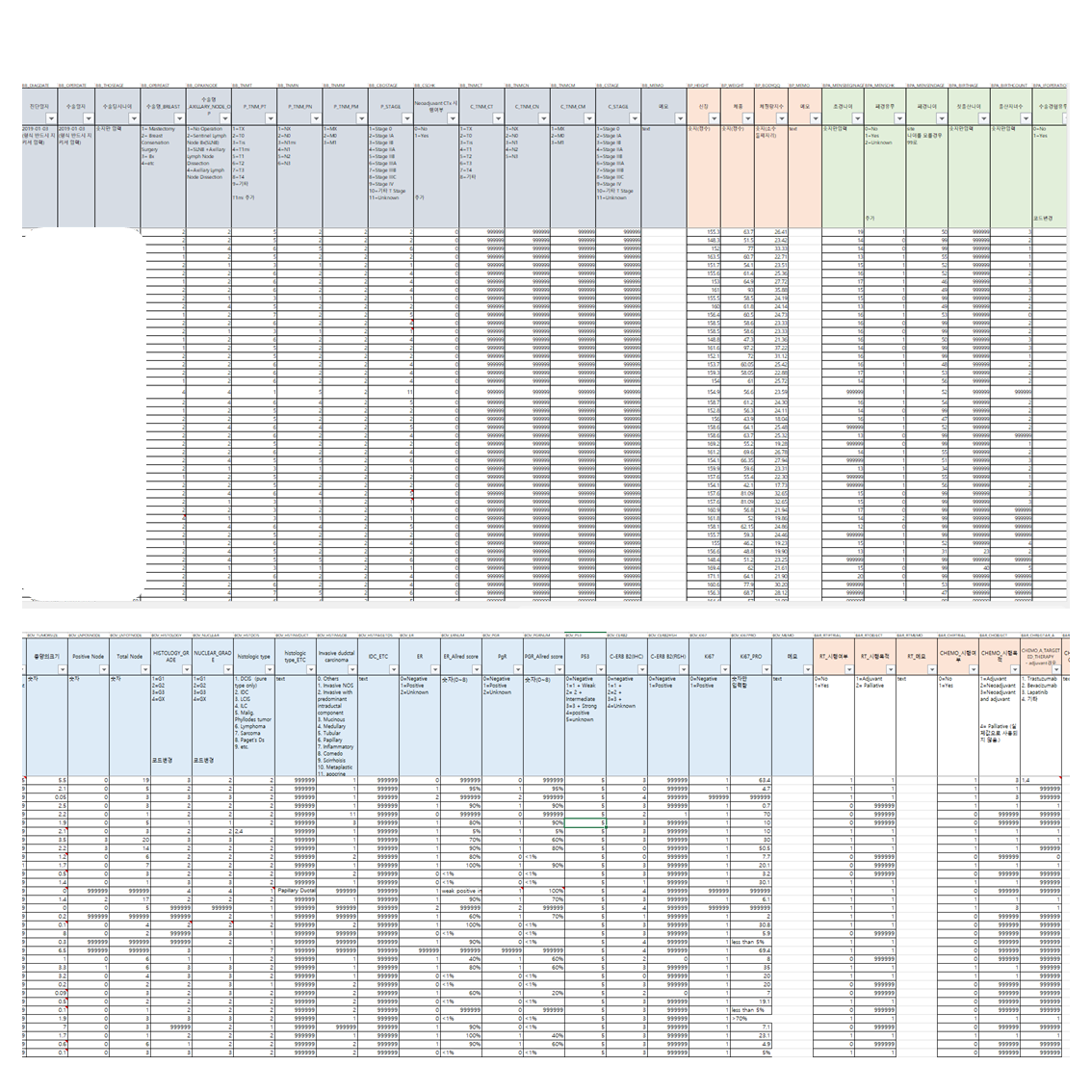

Supplement: Multimedia Appendix 1 [file medinform-v13-e73605-s001.png]

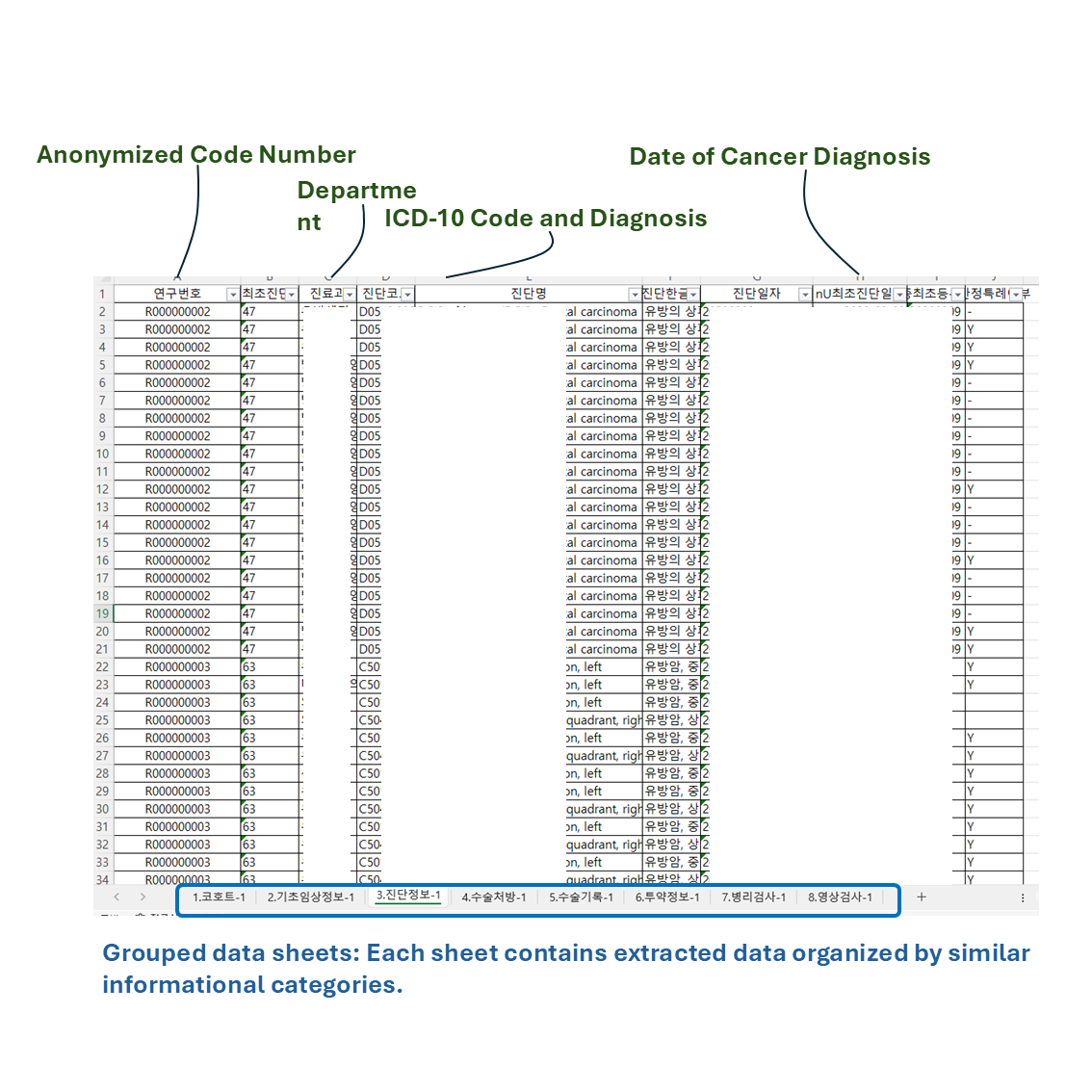

Supplement: Multimedia Appendix 2 [file medinform-v13-e73605-s002.png]

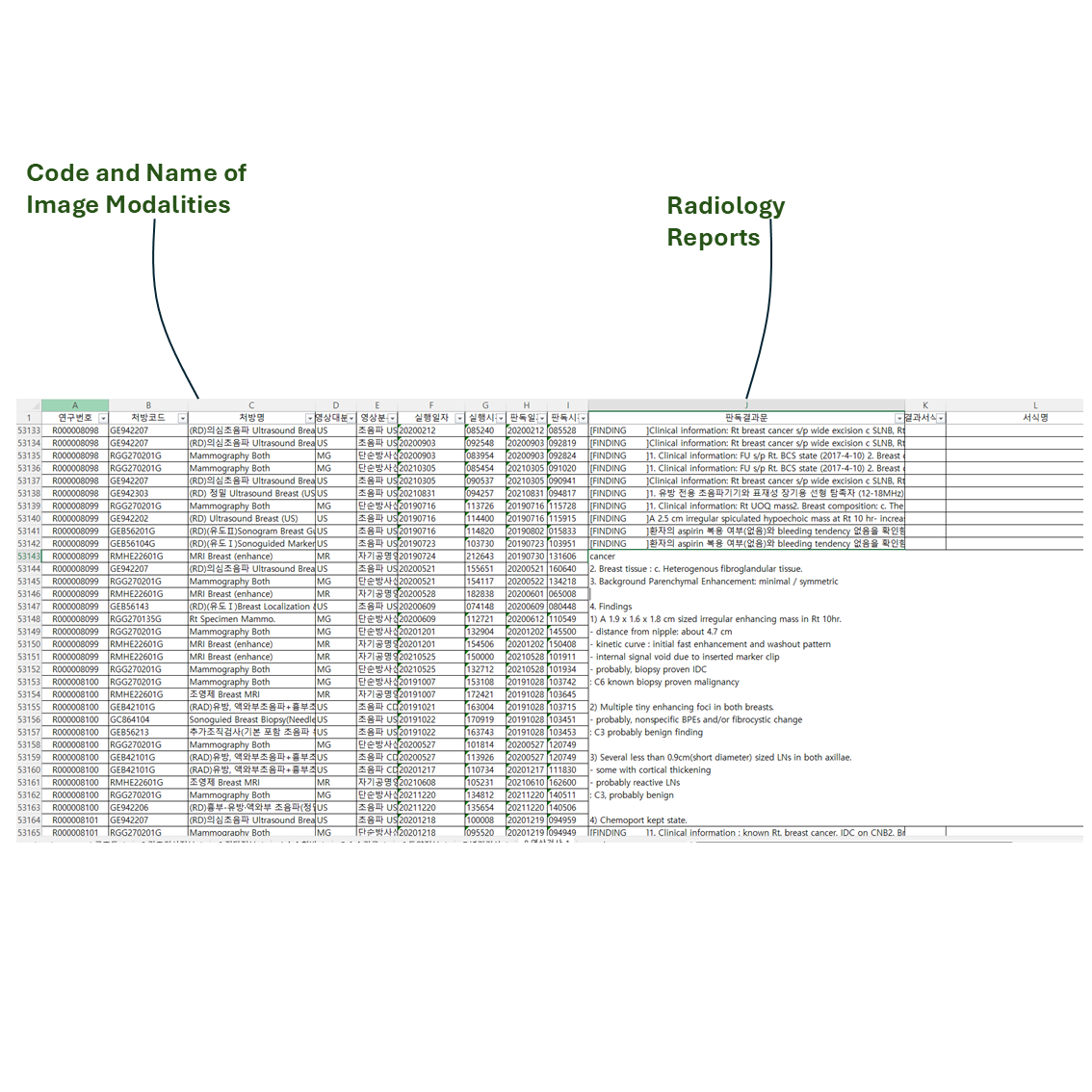

Supplement: Multimedia Appendix 4 [file medinform-v13-e73605-s004.png]
