# Supplementary material for: Large Language Model Versus Manual Review for Clinical Data Curation in Breast Cancer: Retrospective Comparative Study
Source: JMIR Med Inform. 2025 Nov 6;13:e73605. doi: 10.2196/73605 (PMC12599480; doi:10.2196/73605)
Supplement: Multimedia Appendix 5 [file medinform-v13-e73605-s005.docx]

This prompt is a condensed synopsis of the prompts utilized. The prompts were categorized by subject area, with examples incorporated for enhanced comprehension.

# Breast Cancer Clinical Data Extraction Protocol

## 1. Global Processing Rules

### 1.1 Data Format Standards

- Patient ID Format: R[9-digit number]

- Laterality: RT/LT/Bilateral

- Date Format: YYYY-MM-DD

- Missing Data: Code as 999999

- Output Format: CSV, comma-delimited

- Sort: By ID number ascending

### 1.2 File Organization

- Process each case by laterality

- Format: Case_ID = Patient_ID_Laterality

- Example: R000000001_RT

## 2. Clinical Data Processing

### 2.1 Diagnosis Information

1. Source Priority:

- Primary diagnosis information from both diagnosis text and ICD-10 codes

- English diagnosis text only

- Remove Korean text

2. Multiple Diagnosis Handling:

- Diagnosis Deduplication Rule: Compare ICD-10 codes using first three characters (letter + first 2 digits)

Example: C50.1, C50.2, C50.9 → all considered same diagnosis (C50)

D05.1, D05.7 → same diagnosis (D05)

C50.x and D05.x → different diagnoses, keep both

- Same diagnosis (same 3-character prefix): Keep only the earliest date

Example: C50.1 on 2019-01-05 and C50.9 on 2019-03-10 → Keep 2019-01-05

- Different diagnoses (different 3-character prefix): Keep each diagnosis with its own date

Example: C50 (invasive) on 2019-02-01 and D05 (DCIS) on 2019-01-15 → Keep both

- Calculate age at diagnosis for each retained diagnosis

3. Output Format:

Case_ID, Diagnosis_Date, ICD10_Code, English_Diagnosis, Age_at_Diagnosis

- R000000001_RT, 2019-01-15, D05, Ductal carcinoma in situ, 52

- R000000001_RT, 2019-02-01, C50, Invasive carcinoma of breast, 52

### 2.2 Basic Clinical Information

1. Data Selection:

- Use data closest to diagnosis date

- BMI calculation required

2. Required Fields:

- Height (cm)

- Weight (kg)

- BMI (calculated)

- Additional measurements

### 2.3 Surgical Information

1. Processing Rules:

- Base on prescription date

- Prioritize cancer procedures

- List multiple procedures horizontally

2. Surgery Types:

- Excision

- Wide excision

- MRM

- Lumpectomy

- Additional procedures (SLNB/ALND)

### 2.4 Mortality Data

1. Required Fields:

- Mortality Status (N/Y)

- Date of Death (if applicable)

- Last Follow-Up Date

2. Processing Rules:

- Cut-off date: 2023-10-31

- Clear death dates after cut-off

- Update status accordingly

## 3. Pathology Data Extraction

### 3.1 Section Priority

1. MICROSCOPIC DESCRIPTION

2. DIAGNOSIS

3. IMMUNOHISTOCHEMISTRY

4. GROSS DESCRIPTION (size only if needed)

### 3.2 Tumor Assessment

1. Type Identification:

- Invasive carcinoma

- DCIS

- Mixed (invasive + DCIS)

- Microinvasive

- Note secondary components

2. Size Determination:

Priority Search Terms:

a) "Tumor size(size of largest invasive carcinoma);"

b) "Greatest dimension of largest invasive focus:"

c) "size of largest invasive focus;"

d) "Estimated Size (Extent) of DCIS;"

EXCLUSION RULES - Do NOT use:

Gross specimen size (e.g., "Specimen measures 5.0 x 4.0 x 3.0 cm")

Total excision size

Skin ellipse dimensions

Margin measurements (e.g., "deep margin 0.3 cm")

Breast tissue dimensions

Any measurement preceded by "specimen" or "excision"

Valid Examples:

✓ "Tumor size: 2.3 cm"

✓ "Invasive carcinoma measuring 1.8 cm"

✗ "Specimen: 5.0 x 4.0 cm"

✗ "Excised tissue measures 8.0 cm"

3. Grade Recording:

- Invasive: Nottingham (1-3)

- DCIS: Nuclear (1-3)

- Include component scores

### 3.3 Lymph Node Status

1. Data Location Priority:

- Section "j)" or "Lymph Node metastasis"

- DIAGNOSIS entry

- TNM Classification

2. Extraction Patterns:

Format: X/Y (positive/total)

Examples:

- "metastatic carcinoma (n/total)"

- "LN metastasis; present/absent (n/total)"

- "sentinel/axillary LN; n/total"

3. Classification:

- Macrometastasis (>2mm)

- Micrometastasis (0.2-2mm)

- ITC (≤0.2mm)

### 3.4 Biomarker Profile

1. Hormone Receptors (ER/PR):

- Format: Status(Score/Percentage)

- Examples:

Allred score: Pos(8) or Neg(0), range 0-8

Percentage: Pos(95%) or Neg(<1%), range 0-100%

- Validation:

Allred: 0-8 range

Percentage: 0-100% range

If both reported, prioritize Allred score

2. HER2 Assessment:

Initial IHC Score: 0, 1+, 2+, 3+

If 2+: Check for ISH (FISH/SISH/CISH)

Final Status Classification:

Positive: 3+ by IHC OR 2+ with ISH amplification (ratio ≥2.0)

Negative: 0 or 1+ by IHC OR 2+ with ISH non-amplified (ratio <2.0)

Equivocal: 2+ with ISH pending or not performed

3. Ki-67 Index:

- Format: Whole number percentage

- Valid range: 0-100%

## 4. Radiology Data Processing

### 4.1 Image Selection

1. Timing Rules:

- Pre-surgery only

- Within 7 months

- Flag if >7 months

2. Multiple Studies:

- Use earliest qualified study

- Document multiple lesions

### 4.2 Required Measurements

1. Size Documentation:

- Mammogram (mm)

- Ultrasound (mm)

- MRI (mm)

- Convert all to mm

2. Additional Features:

- BI-RADS category

- Density classification

- Lesion characteristics

### 4.3 Ultrasound Specific

1. Lesion Priority:

```

C6 > C5 > C4c > C4b > C4a

```

2. Size Rules:

- Use longest dimension

- Ignore descriptive measurements

- Document multiple lesions

## 5. Quality Control

### 5.1 Required Validations

1. Data Completeness

2. Range Checks

3. Logic Validation

4. Cross-reference Verification

### 5.2 Common Errors

1. Size Measurements:

- No gross specimen sizes

- No excision dimensions

- No margin measurements

- No lymph node sizes

- Use only actual tumor/lesion measurements

2. Node Counts:

- No double counting

- Verify totals

- Check staging consistency

3. Biomarkers:

- Range validation

- Logic checks

- Required follow-up

### 5.3 Documentation

1. Missing Data:

- Reason required

- Alternative sources checked

- QC flags added

2. Special Cases:

- Multiple specimens

- Bilateral cases

- Neoadjuvant therapy
