# Supplementary material for: Large Language Model Versus Manual Review for Clinical Data Curation in Breast Cancer: Retrospective Comparative Study
Source: JMIR Med Inform. 2025 Nov 6;13:e73605. doi: 10.2196/73605 (PMC12599480; doi:10.2196/73605)
Supplement: Checklist 1 [file medinform-v13-e73605-s006.pdf]

TRIPOD-LLM Checklist

Item	Description	Section	Status
**TITLE AND ABSTRACT**			
1a	Identify study as developing/validating LLM model	Title section	✓
2	Structured abstract with key information	Abstract section	✓
**INTRODUCTION**			
2	Background and rationale	Introduction section	✓
3a	Study objectives	Introduction section, final paragraph	✓
3b	Specify predictive vs explanatory	N/A	Not applicable
**METHODS**			
4a	Describe data sources	Methods - Study Design and Data Collection	✓
4b	Study setting and key dates	Methods - Study Design	✓
5a	Participant eligibility criteria	Methods - Study Design	✓
5b	Participant recruitment method	Methods - CDW query	✓
6a	LLM model name and version	Methods - Data Curation section	✓
6b	Model access date	Methods - Prompt Development	✓

6c	API access method and specifications	Methods - LLM Implementation and API Access	✓
7a	Prompt development process	Methods - Prompt Development Process	✓
7b	Final prompt specification	Methods - Supplementary Prompt	✓
8	Input data preprocessing	Methods - LLM Implementation	✓
9a	Output format specification	Methods - Task Sequence and Processing Flow	✓
9b	Post-processing procedures	Methods - Quality Control Module	✓
10a	Define all outcomes	Methods - Objectives and Statistical Analysis	✓
10b	How outcomes were assessed	Methods - Data Quality Assessment and Validation	✓
11	Sample size determination	N/A	Not applicable
12a	Statistical analysis methods	Methods - Objectives and Statistical Analysis	✓
12b	Data privacy and security measures	Methods - Ethical Considerations	✓
12c	Missing data handling	Methods - Quality Control Module	✓
**RESULTS**			
13a	Participant flow and numbers	Results - Outcomes	✓
13b	Reasons for exclusion	N/A	Not applicable
14a	Baseline characteristics	Results - Table 2	✓
14b	Missing baseline data	Results - Figure 2	✓
15	Primary outcome results	Results - Validation analysis	✓
16	Model performance metrics	Results - Outcomes (90.8% accuracy)	✓
17	Resource utilization (time, cost)	Results - Processing Time and Resource Utilization, Table 1	✓
**DISCUSSION**			
18	Summary of key findings	Discussion - Principal Findings	✓
19	Study limitations	Discussion - Strengths and Limitations	✓
20	Clinical interpretation	Discussion - Comparison to Prior Work	✓
21	Generalizability	Discussion - Strengths and Limitations (Fourth limitation)	✓
22	Implications for practice/research	Discussion - Future Directions	✓

OTHER INFORMATION

23	Study registration	N/A	Not applicable
24	Study protocol availability	Methods section + Supplementary Figures	
25	Code availability	Code Availability section	✓
26	Data availability	Data Availability Statement	✓
27	Conflicts of interest	Competing interests	✓
28	Funding	Funding section	✓

LLM-Specific Items

S1	Temperature settings	N/A (default settings used)	Not applicable
S2	Token limits and handling	Methods - LLM Implementation (Input size limitations)	✓
S3	Reproducibility measures	Methods - Prompt Development Process (fixed seed)	✓
S4	Error handling procedures	Methods - LLM Implementation (Quality control)	✓
S5	Prompt versioning	Supplementary File 2	✓
S6	API cost reporting	Results - Table 1 ($260)	✓
